# Evaluation of the Chagas VirClia^®^ and Chagas TESA VirClia^®^ for the Diagnosis of *Trypanosoma cruzi* Infection

**DOI:** 10.3390/pathogens12010050

**Published:** 2022-12-28

**Authors:** Isabel García-Bermejo, David Molina Arana, Gloria Zaragoza Vargas, Blanca Carrasco Fernández, Emilia García, Javier Nieto, Maria Delmans Flores-Chávez

**Affiliations:** 1 Microbiology Department, Hospital Universitario de Getafe, 28905 Madrid, Spain; 2 Leishmaniasis and Chagas Disease Unit, National Centre for Microbiology, Instituto de Salud Carlos III, 28220 Madrid, Spain; 3 Mundo Sano Foundation, 28002 Madrid, Spain

**Keywords:** Chagas disease, *Trypanosoma cruzi* infection, serological diagnosis, sensitivity, specificity, chemiluminescence immunoassay, recombinant antigens, TESA

## Abstract

Chagas disease (CD), caused by the protozoan *Trypanosoma cruzi*, is an important problem of public health even in regions where it is not endemic. Spain ranks second worldwide in terms of imported cases of *T. cruzi* infection in the chronic phase. The diagnosis in this stage is made via the detection of antibodies against *T. cruzi*. Therefore, we aimed to evaluate the sensitivity and specificity of two fully automated chemiluminescence immunoassays, Chagas VirClia^®^ (CHR), which uses a mixture of recombinant antigens, and Chagas TESA VirClia^®^ (TESA), the first chemiluminescence assay based on excretion-secretion antigens of trypomastigotes, both designed in monotest format. A retrospective case–control study was performed using 105 well-characterized samples: 49 from patients with CD, 22 from uninfected individuals, and 32 from patients with other pathologies. Sensitivity was 98% for CHR and 92% for TESA. In contrast, the specificity in both was 100%. Cross-reactivity was observed in leishmaniasis (2/10). CHR meets the criteria to become a tool for serological screening, while TESA has the potential for confirmation and cross-reaction discrimination. The monotest format allows its application in laboratories with a small number of samples. The high specificity of both assays is useful in areas where leishmaniasis is endemic.

## 1. Introduction

Chagas disease (CD), also known as American trypanosomiasis, is caused by infection with the protozoan parasite *Trypanosoma cruzi* (*T. cruzi*). It is estimated that 6 to 7 million people are infected worldwide, mainly in Latin America [1].

The infection is characterized by an acute, often asymptomatic stage where active parasitemia is evident. If left untreated, the infection enters a chronic phase and most individuals remain asymptomatic, but after years or decades, 30–40% of them progress to an advanced symptomatic form with potentially life-threatening cardiac, gastrointestinal, or other complications [2,3]. Infected individuals with chronic CD represent a substantial population capable of transmitting the infection [4,5].

CD is clinically curable if treatment is initiated at an early stage. Therefore, universal access to prompt diagnosis and care is essential [1,2,3].

Once totally confined to the region of the Americas, where it is mostly triatomine-vector-borne, CD has spread to other continents over the last century, mainly because global population movements mean that it can be located wherever migrants from endemic areas settle. Migrant populations and some modes of transmission such as blood and organ donation, bone marrow transplantation, congenital transmission, and tainted foods and fluids have caused the spread of this disease beyond its natural geographical boundaries [6]. Spain ranks second worldwide in terms of imported cases of *T. cruzi* infection, only surpassed by the United States [7,8,9]. In this country (Spain), the population at risk of suffering from the disease is predominantly asymptomatic, undergoing the chronic phase of the disease and many of them are in post-treatment follow-up.

The diagnosis of CD depends on the phase of infection. During the acute phase of infection, or reactivation due to immunosuppression, direct methods and PCR are the main diagnostic tools used for *T. cruzi* detection. Nevertheless, these approaches show low sensitivity during the chronic phase of infection, when the parasite is hidden in target tissues, and diagnosis relies upon the detection of *T. cruzi* antibodies by serological methods [2]. In this phase, parasitological techniques are usually negative and PCR tests show variable sensitivity (50–80%) [10,11]; PCR performance depends on the presence of the parasite in blood and is currently not considered efficient for chronic CD diagnosis [12]. PCR tests in the chronic phase are focused on therapeutic failure detection [13].

Serology is the method of choice to confirm the diagnosis of CD and to screen the population at risk from endemic areas (blood donors, pregnant women, organ donors). The serological diagnosis is hampered by the lack of a gold standard and the availability of multiple types of assays with varying sensitivity and specificity. The differential diagnosis for positive *T. cruzi* serology entails cross reactivity with other pathogens (i.e., *Leishmania spp*., *Plasmodium spp*., and *Trypanosoma rangeli*) or autoimmune diseases. For this reason, for the diagnosis of *T. cruzi* infection, the World Health Organization (WHO) still recommends using two serological tests based on different antigens and/or principles, performed in parallel to reduce the risk of false positive results [2,14,15]. In case of discordant results, a serological examination must be repeated in a new sample, and if results remain inconclusive, a confirmatory test should be performed. The most commonly used serological methods are enzyme-linked immunosorbent assay (ELISA) and immunofluorescent indirect test (IFI) [15]. However, the serological techniques to be used for the diagnosis and screening of CD continue to evolve. To increase sensitivity and specificity, new serological methods that use recombinant antigens or synthetic peptides, as a replacement for conventional tests based on total antigens, have been developed.

In the last decade, a few high-throughput automated systems based on chemiluminescence have been introduced and the results of their performance show they have a potential for use as a single assay for screening and diagnosis of chronic CD [16,17,18]. Recently, two fully automated chemiluminescence immunoassays in monotest format were developed. Chagas VirClia^®^ uses a mixture of recombinant antigens, and Chagas TESA VirClia^®^ uses excretion-secretion antigens of trypomastigotes for the first time in an automated assay. Both assays are designed for the qualitative detection of IgM and IgG antibodies against *T. cruzi*. Considering these characteristics and the CD situation in Spain, we aimed to assess the sensitivity and specificity of these assays to screen and confirm *T. cruzi* infection, respectively, using well-characterized samples.

## 2. Materials and Methods

### 2.1. Study Design and Sample Description

A retrospective case-control model was performed, selecting samples for convenience from the serum collection of the Getafe University Hospital (Getafe, Madrid, Spain) and the National Centre for Microbiology (CNM, Spanish acronym), Instituto de Salud Carlos III (Majadahonda, Madrid, Spain). A panel of 105 serum samples was assembled, which consisted of 49 samples from CD patients, 22 from uninfected individuals, 10 from malaria patients, 10 from patients with visceral leishmaniasis (VL), 4 with acute Epstein–Barr virus infection, 4 with acute cytomegalovirus infection, 4 with toxoplasmosis, and 2 with autoimmune diseases. The eligibility criteria were based on previous results by referral tests of each pathology and their volume availability.

### 2.2. Reference Diagnostics Tests

The categorization of the sera regarding CD and the reference diagnosis of CD was based on the combination of the results of a conventional ELISA in-house (ELISA-CNM), a non-conventional ELISA (Chagatest recombinante v 4.0, Wiener, Argentina), and an indirect immunofluorescence (IFI-CNM). All these tests determined anti-*T. cruzi* IgG antibodies against total (ELISA and IFI-CNM) or recombinant antigens (recombinant Chagatest) and were carried out according to Flores-Chavez et al. [19] and manufacturer’s instructions, respectively.

### 2.3. Index Diagnostic Tests

Two different chemiluminescence techniques were used: Chagas VirClia^®^ (recombinant VirClia), which uses a mixture of recombinant antigens, and Chagas TESA VirClia^®^ (TESA VirClia), which uses excretion-secretion antigens of trypomastigotes. Both assays were designed for the qualitative detection of specific IgM and IgG antibodies against *T. cruzi* in monotest format and must be performed on the VirClia^®^ automatic system (Vircell^®^, Spain). The monotest format includes all the necessary quality controls with no need for extra controls or calibration. Both immunoassays were performed following the manufacturer’s instructions. Results of the chemiluminescence reaction were measured in relative luminescence units (RLU) and expressed as sample RLUs/cut-off value (S/CO) as follows: positive (S/CO > 1, 1); grey zone (S/CO between 0, 9–1, 1), and negative (S/CO < 0, 9).

### 2.4. Data Analysis

Sensitivity, specificity, and confidence interval values (CI) were calculated using the binomial distribution analysis. The differences concerning the reference diagnosis were estimated using McNemar’s test and considering a *p* < 0.05 as statistically significant.

## 3. Results

### 3.1. Reactivity and Performance of Chagas VirClia^®^ and Chagas TESA VirClia^®^

**Figure 1:**
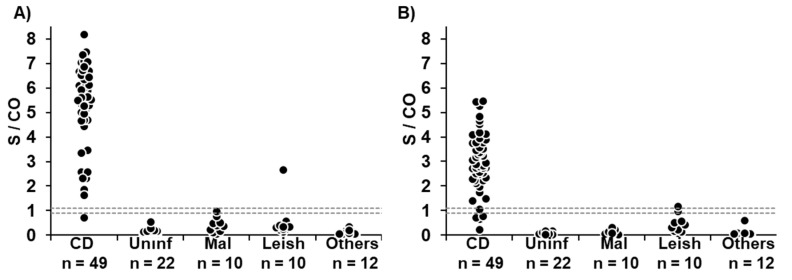
Levels of anti-*T. cruzi* antibodies by: (**A**) Chagas VirClia^®^ and (**B**) Chagas TESA VirClia^®^. CD, Chagas disease patients; Uninf, uninfected individuals; Mal, malaria patients; Leish, visceral leishmaniasis patients; Others, individuals with other pathologies. Dashed lines set the limits of the gray zone.

Out of 49 CD patients, 48 and 45 cases returned positive results by recombinant and TESA VirClia tests, respectively. Therefore, the sensitivity of the recombinant test was higher (98%) than the TESA test (92%); however, this difference was not statistically significant (McNemar’s test = 2.250, *p* = 0.125), nor were the results significant for the case definition (recombinant VirClia vs case definition: McNemar’s test = 0, *p* = 1; TESA VirClia vs. case definition: McNemar’s test = 3.2, *p* = 0.063). In contrast, all 22 samples from uninfected individuals returned negative results by both approaches, so the specificity was 100% (Table 1, Figure 1).

### 3.2. Agreement Analysis

In qualitative terms, in samples from the CD population at risk, recombinant and TESA VirClia showed a kappa index of 0.97 (*p* < 0.001) and 0.94 (*p* < 0.001), respectively, with respect to composite case definition. Discrepant results in five CD samples were related to low reactivity by IFI (Table 2).

### 3.3. Cross Reactivity with Other Pathologies

To assess the cross-reactivity issues, 32 samples of individuals with other pathologies were included in the study. Out of 10 samples from VL patients, 1 sample returned a positive result by recombinant VirClia (case Leish 13, Table 2, Figure 1A) and another by TESA VirClia (case Leish 14, Table 2, Figure 1B). The case Leish 13 yielded an S/CO value within the grey zone by TESA VirClia (Table 2).

Out of 10 samples from malaria patients, 9 returned undoubtedly negative by both VirClia tests, and 1 sample within the grey zone value by recombinant VirClia but negative by TESA VirClia. All 12 remaining samples from other pathologies returned negative results by both VirClia tests (Figure 1).

## 4. Discussion

Many serological techniques have been developed for the detection of specific *T. cruzi* antibodies. Crude antigen-based tests, including the indirect hemagglutination assay (IHA), IFI, and ELISA, have been widely used in the past. As expected, these tests lacked specificity due to frequent cross-reactions with other protozoa antigens. The development of techniques using recombinant antigens and chimeric recombinant antigens [20] has helped to overcome the previous problem of specificity as well as to improve the sensitivity of the serological tests. The highly sensitive and specific new assays for the detection of anti-*T. cruzi* antibodies could help to reduce expenses for additional second-line testing for the diagnosis and prevention of the disease. Other advantages of new-generation tests are automation, rapidity, and high performance. Detection systems such as chemiluminescence increase light amplification and signal duration in comparison with traditional ELISAs.

In the present study, the recombinant VirClia assay demonstrated good analytical sensitivity and suitable specificity compared to established assays and supports its use as a screening test. This chemiluminescent assay contains a mixture of different recombinant *T. cruzi* antigens: the major antigenic epitopes of cytoskeletal-associated protein (FRA), a trypomastigote surface protein (B13), and a recombinant MACH multi-antigenic protein including PEP2, TcD, TcE, and SAPA antigens, which allow obtaining high sensitivity without losing specificity. The negative result observed in patient CD2 (Table 2) by the recombinant VirClia was related to antibody levels with low reactivity in the reference techniques. This sample belonged to a patient in follow-up after trypanocidal treatment, but as this intervention was carried out in their country of origin, we do not have sufficient certainty of the scheme and its compliance, and therefore we did not rule it out at the time of sample selection. On the other hand, although we could not verify the loss of reactivity due to the freeze/thaw process, this fact could have affected the final result of this sample. Discarding the result of patient CD2 from the global analysis, recombinant VirClia would have a diagnostic sensitivity of 100%. Interestingly, only one serum sample of the 10 patients diagnosed with leishmaniasis was reactive in the recombinant VirClia assay (Leish 13, Table 2). Regarding TESA VirClia, which uses excretion-secretion antigens of trypomastigotes, its sensitivity was somewhat lower; however, this difference was not statistically significant. Like recombinant VirClia, all serum samples from uninfected individuals were non-reactive by TESA VirClia, making both assays useful for ruling out the infection by the *T. cruzi* parasite.

The use of a mixture of recombinant antigens in combination with signal amplification by chemiluminescence system supposed a higher accuracy in the diagnosis of CD in comparison with conventional techniques used in this study. Other advantages of both chemiluminescence immunoassays are that the presentation in monotest format allows performing the assay individually without the need to group samples, all reagents are ready-to-use and include the quality controls.

The main limitation of this study is the sample size. A systematic review of the prevalence of CD in Latin American migrants in Europe found an estimated overall prevalence of 4.2%, although considerable heterogeneity was found between and within different migrant groups [21]. People from Bolivia and Paraguay had the highest prevalence of CD, with 18.1% and 5.5%, respectively, whereas the prevalence among migrants from other countries such as Brazil, Peru Colombia, and Venezuela was around or less than 1%.

According to the National Statistics Institute of Spain, the number of Latin American immigrants living in Madrid (Spain) is 211,123 [22]. The Hospital Universitario de Getafe is located in Getafe, a municipality south of Madrid, and serves a population of 183,095 inhabitants, of whom 13,658 were born in CD-endemic areas [23]. All individuals at risk living in this area could be attended at the hospital or primary care centers and *T. cruzi* serology should be accessible. In this geographic area, the prevalence of CD is low (1.2%), and out of the total immigrant population, those from Bolivia and Paraguay are a minority, at 1.2% and 0.94%, respectively. This population generally suffers from chronic CD or is being monitored after treatment, so the parasite load is low, unlike what occurs in acute infections more typical of countries where the disease is endemic. Therefore, in these situations, it is important to diagnose the infection but also to exclude it. In this sense, both recombinant and TESA VirClia are useful.

The Pan American Health Organization (PAHO) recently published new guidelines for diagnosis and treatment, Chagas Disease with Grading of Recommendations Assessment, Development and Evaluation (GRADE), mainly to aid the management of CD in endemic areas [15]. However, it is also important to address the problem in non-endemic areas such as Europe [24]. Considering the characteristics of endemic and non-endemic countries, different strategies could be implemented to improve the care of CD patients [25].

As the diagnostic strategy for CD varies according to location and purpose, one of the main challenges in Spain is underdiagnosis, despite evidence suggesting the cost-effectiveness of screening [26]. The suitability of commercial tests in non-endemic areas such as Spain, with other endemic protozoans that can cause cross-reactivity, is also a factor to be taken into account. Serodiscordance in CD remains a challenge since individuals with inconclusive results are clinically complicated to manage. Different reasons may lead to repeatedly discordant results such as the immune response host heterogeneity, test-dependent factors, and cross-reactions with other pathogens. In addition, *T. cruzi* is a heterogeneous species with a wide genetic diversity, which has been grouped by consensus into seven discrete typing units (DTUs) affecting humans. Data suggest that the sensitivity of serologic assays varies by geographic localization, possibly due to *T. cruzi* DTUs differences and resulting antibody responses [27]. However, a study carried out in Spain, in a population of Latin American immigrants (mostly Bolivians), has shown that there is no correlation between serological titers and the different DTU of the parasite [28]. Another cause of serodiscordance could be the drop in anti-*T. cruzi* antibody levels after treatment [29], although this phenomenon occurs for a long time. Without knowledge of this factor, tests with particular antigen as TESA VirClia could return negative results. More studies are needed to evaluate its potential for tripanocidal post-treatment follow-up.

The implementation of screening and diagnosis in populations at risk wherever they live, such as in Spain, will improve the appropriate intervention to mitigate CD.

## 5. Conclusions

According to our study, Chagas VirClia^®^ meets the criteria to become a tool for serological screening, while Chagas TESA VirClia^®^ has the potential as a resource for confirmation and cross-reaction discrimination. This approach simplifies routine diagnosis of infection and reduces costs in the laboratory. The presentation in monotest format allows its application in laboratories with a small number of samples. The high specificity of both assays is useful in areas where leishmaniasis is endemic.

## Figures and Tables

**Table 1:** Estimation of sensitivity and specificity of Chagas VirClia^®^ and Chagas TESA VirClia^®^ by binomial distribution analysis.

Test	Infection Status		Sensitivity		Specificity	
	Infected	Uninfected	%	IC 95%	%	IC 95%
**Recombinant VirClia**						
Positive	48	0	98 *	89–100	100	84.6–100
Negative	1	22				
**TESA VirClia**						
Positive	45	0	92 *	80–98	100	84.6–100
Negative	3	22				

* According to McNemar’s test, the differences between monotests were not significant (McNemar’s test = 2.250, *p* = 0.125), nor were tests for the case definition (recombinant VirClia vs case definition: McNemar’s test = 0, *p* = 1; TESA VirClia vs case definition: McNemar’s test = 3.2, *p* = 0.063).

**Table 2:** Samples with discrepant results.

Status Condition	Code	Recombinant VirClia 1.1 *	TESA VirClia 1.1 *	IFA-CNM 1/40 *	ELISA-CNM 1.2 *	Recombinant Chagatest 1 *
CD ^1^	1	1.868	0.239	1/20	1.200	1.670
CD	2	0.712	0.666	1/80	5.440	1.700
CD	3	3.460	0.728	1/80	3.110	6.900
CD	4	1.633	0.777	1/80	2.620	3.420
CD	5	3.369	1.053	1/80	2.840	7.349
Malaria	6	0.970	0.081	1/20	0.429	0.401
Leish ^2^	7	0.239	0.181	>1/160	2.621	0.206
Leish	8	0.325	0.225	>1/160	2.433	0.501
Leish	9	0.274	0.331	>1/160	2.968	0.681
Leish	10	0.576	0.450	>1/160	5.037	0.733
Leish	11	0.346	0.509	>1/160	2.984	0.687
Leish	12	0.423	0.587	>1/160	3.446	0.674
Leish	13	2.668	0.978	>1/160	7.132	4.323
Leish	14	0.351	1.174	>1/160	3.753	1.230

* Threshold of each test; ^1^ CD, Chagas disease; ^2^ Leish, visceral leishmaniasis

## Data Availability

Raw data are available by writing to the corresponding author.
